# Robert C. Coffey Reviews the Work of Clark and of Yates Regarding the Mechanical Principles Involved in Perineal Drainage

**Published:** 1907-04

**Authors:** 


### ROBERT C. COFFEY REVIEWS THE WORK OF CLARK AND OF YATES REGARDING THE MECHANICAL PRINCIPLES INVOLVED IN PERINEAL DRAINAGE.

---

From this and the author's own experience, he draws the following deductions and conclusions:

Fluids are rapidly absorbed by all parts of the peritoneum: crude or granular matter is absorbed only through the lymph spaces at the diaphragm. (Clark.)

A drainage tract always contains mico-organisms. (Clark.)

Any form of drain is isolated from the free peritoneal cavity in few hours. (Yates.)

A drain causes a flow of serum, by mechanical irritation, which is profuse in proportion to the amount of drainage material in contact with peritoneum. (Yates.)

This flow is delivered to the surface only in proportion to the amount of drainage passing out through the wound. (Experiment.)

Serum and pus accumulate around drainage only when the drainage at the outlet is less in amount than that contained inside or when drainage is too small. (Experiment and clinical observation.)

Blood and pus in quantity are never found in the neighborhood of a properly applied drain after forty-eight hours. (Clinical observation.)

The flow of serum resulting from the irritation of a drain in the free peritoneal cavity dissolves blood clots and thick pus, leaving gauze drainage clean and white by the time the flow ceases. (Experiment and clinical observation.)

Drainage placed within a walled abscess cavity does not excite a flow of serum, therefore blood and pus are not drained from formed abscess cavities by capillary action. (Experiment and clinical observation.)

Small gauze drains are likely to be choked at the exit unless covered by a tube or protective. (Clinical observation.)

Extensive gauze drains are not thus affected and act in proportion to the amount coming through the opening, but will only drain when it is in contact with dressing or clothing, or when its outside end is not at a lower level than the depth of the cavity to be drained. (Experiment and clinical observation.)

A tubular drain drains perfectly a walled cavity or will drain the peritoneal cavity under the influence of gravity if it does not become choked, but will not drain up hill, except when the fluid is confined. (Experiment and clinical observation.)

A single tube is much more likely to be choked by intestines and omentum than two or more parallel tubes. (Clinical observation.)

There are three cavities or basins of the peritoneum to be drained; the right and left flanks, separated from each other by the spinal column, and the pelvis, separated from the flanks by the psoas muscles. Either flank holds more fluid and is an inch deeper than the pelvis and its bottom is more than four inches below the top of the divide made by the psoas muscle on which the appendix rests. The body must be elevated to an angle of fifty-one degrees to bring the bottom of the flank on a level with the divide, and to an angle of sixty or seventy degrees to properly drain by the Fowler position. The entire abdominal cavity can be drained by a lateral position.

A line drawn from the center of the surface of the perineum to the tip of the shoulder will pass through the deepest part of the pelvis and flank. I have called these lines the right and left drainage lines.

There are no independent cavities or basins between these lines. As it is a well-known clinical fact that drainage should always be in contact with the parietal peritoneum on at least one side, I make it a general rule that drainage should never be placed between these drainage lines except just above the pubic bones. The deepest points of the flank are best drained external to these lines (cadaver and plaster cast).

Gravity is the most important principle in peritoneal drainage, therefore drainage must reach the most dependent point of the cavity to be drained. The patient must be placed in the position that would naturally cause the fluids in the peritoneal cavity to gravitate to the drain, always bearing in mind the anatomy of the three anatomical cavities or basins.

Gauze or capillary drainage is the most widely applicable and useful of all drains if used in sufficient quantity to preclude its being choked by debris and provided the drain is as large in circumference at its exit as it is at any point within the cavity, and provided it is in contact with abundance of dressings on the outside.

Gauze drainage is a very dangerous agent if all the foregoing principles are not kept in mind in its application.

If a surgeon remembers that his drain ceases to be effective in a few hours, he places it with the idea of removing septic fluid in the shortest time possible according to the principles of drainage, and usually gets results.

If he is deluded by the belief that his drainage will continue to work for days and that fluid will run up hill to get to the drain, he will usually place his drain accordingly, and is consequently disappointed in drainage.

If a surgeon habitually removes gauze drainage before it is loosened by the natural process, a large per cent. of his cases will have secondary sepsis, post operative obstruction, or post operative hernia.

Drainage (except a small precautionary cigarette or tubular drain) can rarely be safely removed until the fifth or sixth day—many times are best left till the end of 2 or 3 weeks.

Capillary drainage is inefficient for draining defined abscess cavities.

Tubular drainage is appropriate for defined abscess cavities, but is an uncertain drain in the free peritoneal cavity except when aided by gravity.—Journal A. M. A., March 16, 1907.

